# Cohort profile: the Environmental-Pollution-Induced Neurological EFfects (EPINEF) study: a multicenter cohort study of Korean adults

**DOI:** 10.4178/epih.e2021067

**Published:** 2021-09-16

**Authors:** Heeseon Jang, Woojin Kim, Jaelim Cho, Jungwoo Sohn, Juhwan Noh, Gayoung Seo, Seung-Koo Lee, Young Noh, Sung Soo Oh, Sang-Baek Koh, Hee Jin Kim, Sang Won Seo, Ho Hyun Kim, Jung Il Lee, Sun-Young Kim, Changsoo Kim

**Affiliations:** 1 Department of Preventive Medicine, Yonsei University College of Medicine, Seoul, Korea; 2 Department of Public Health, Yonsei University Graduate School, Seoul, Korea; 3 Institute of Human Complexity and Systems Science, Yonsei University, Incheon, Korea; 4 Institute for Environmental Research, Yonsei University College of Medicine, Seoul, Korea; 5 Department of Preventive Medicine, Jeonbuk National University Medical School, Jeonju, Korea; 6 Department of Radiology, Severance Hospital, Yonsei University College of Medicine, Seoul, Korea; 7 Department of Neurology, Gil Medical Center, Gachon University College of Medicine, Incheon, Korea; 8 Department of Occupational and Environmental Medicine, Wonju College of Medicine, Yonsei University, Wonju, Korea; 9 Department of Preventive Medicine, Wonju College of Medicine, Yonsei University, Wonju, Korea; 10 Department of Neurology, Samsung Medical Center, Sungkyunkwan University School of Medicine, Seoul, Korea; 11 Department of Information, Communication and Technology Convergence. ICT Environment Convergence, Pyeongtaek University, Pyeongtaek, Korea; 12 Korea Testing and Research Institute, Gwacheon, Korea; 13 Department of Cancer Control and Population Health, Graduate School of Cancer Science and Policy, National Cancer Center, Goyang, Korea

**Keywords:** Cohort studies, Environmental pollutants, Neurodegenerative diseases, Magnetic resonance imaging, Neuropsychological tests

## Abstract

The general population is exposed to numerous environmental pollutants, and it remains unclear which pollutants affect the brain, accelerating brain aging and increasing the risk of dementia. The Environmental-Pollution-Induced Neurological Effects study is a multi-city prospective cohort study aiming to comprehensively investigate the effect of different environmental pollutants on brain structures, neuropsychological function, and the development of dementia in adults. The baseline data of 3,775 healthy elderly people were collected from August 2014 to March 2018. The eligibility criteria were age ≥50 years and no self-reported history of dementia, movement disorders, or stroke. The assessment included demographics and anthropometrics, laboratory test results, and individual levels of exposure to air pollution. A neuroimaging sub-cohort was also recruited with 1,022 participants during the same period, and brain magnetic resonance imaging and neuropsychological tests were conducted. The first follow-up environmental pollutant measurements will start in 2022 and the follow-up for the sub-cohort will be conducted every 3-4 years. We have found that subtle structural changes in the brain may be induced by exposure to airborne pollutants such as particulate matter 10 μm or less in diameter (PM_10_), particulate matter 2.5 μm or less in diameter (PM_2.5_) and Mn_10_, manganese in PM_10_; Mn_2.5_, manganese in PM_2.5_. PM_10_, PM_2.5_, and nitrogen dioxide in healthy adults. This study provides a basis for research involving large-scale, long-term neuroimaging assessments in community-based populations.

## INTRODUCTION

Dementia—including Alzheimer’s disease (AD), Parkinson’s disease dementia, and vascular dementia—is a global health challenge of increasing importance, as 40-50 million people are currently diagnosed with it [1-3]. This condition also leads to overwhelming national, societal, and familial burdens due to the long course of its progression and demand for continuing care. The global age-standardized disability-adjusted life years (DALY) rate (per 100,000) for AD and other types of dementia has gradually increased from 460.9 (95% confidence interval [CI], 394.5 to 544.4) in 1990, to 469.6 (95% CI, 403.2 to 552.4) in 2006 and 470.6 (95% CI, 401.2 to 556.3) in 2016 [4]. In the United States, the total health care cost for dementia is significantly higher than that for heart disease, cancer, or other illnesses [5], and the total cost for AD care is projected to increase from US$305 billion in 2020 to over US$1 trillion by 2050 [6].

The increasing burden of dementia also represents an issue in Korea. For 2 decades, the Korea has been an aging society and is expected to become a super-aged society in 2025 [7]. A 2014 survey revealed a 9.2% prevalence of dementia among Korean older adults (aged ≥ 65 years); furthermore, the age-specific prevalence of dementia doubled with a 5.8-year increase in age [8]. Dementia contributed the most to the overall disease burden among elderly people in 2010, yielding 274,849 DALYs, an estimate that is projected to increase 3-fold by 2050 [9].

To curb the increasing burden of dementia, prevention is of paramount importance because most types of dementia are progressive and incurable. Specifically, primary prevention strategies involve the identification and management of risk factors. The long-established risk factors for dementia include smoking, heavy alcohol consumption, cardiovascular disease, and diabetes. In recent decades, growing evidence has also emerged regarding environmental pollution (specifically, particulate matter [PM]) as a risk factor for dementia and some early or preclinical phases of dementia (e.g., mild cognitive impairment and cognitive decline). Furthermore, when our cohort was established in 2014, few studies had examined the effect of PM on sensitive neurological outcomes such as brain imaging markers. However, members of the general population are exposed to numerous environmental pollutants in varying degrees, and it remains unclear which pollutants affect the brain, accelerating brain aging and increasing the risk of dementia. Hence, a cohort study specifically designed to comprehensively investigate various environmental pollutants and their effects on the long course of neurodegeneration, from preclinical brain structural changes to overt cognitive decline and dementia, is warranted.

We launched a multi-city prospective cohort study in 2014, named the “Environmental-Pollution-Induced Neurological EFfects (EPINEF)” study, as part of the Environmental Health Action Program of the Korean Ministry of Environment. The aim was to comprehensively investigate the effect of different environmental pollutants on brain structures, neuropsychological function, and the development of dementia in adults. The study may contribute to the improvement of public health policies by presenting guidelines for the management of neurological diseases that reflect the peculiarities of dementia and exposure sectors.

## STUDY PARTICIPANTS

### Study population and design

**Figure 1.**
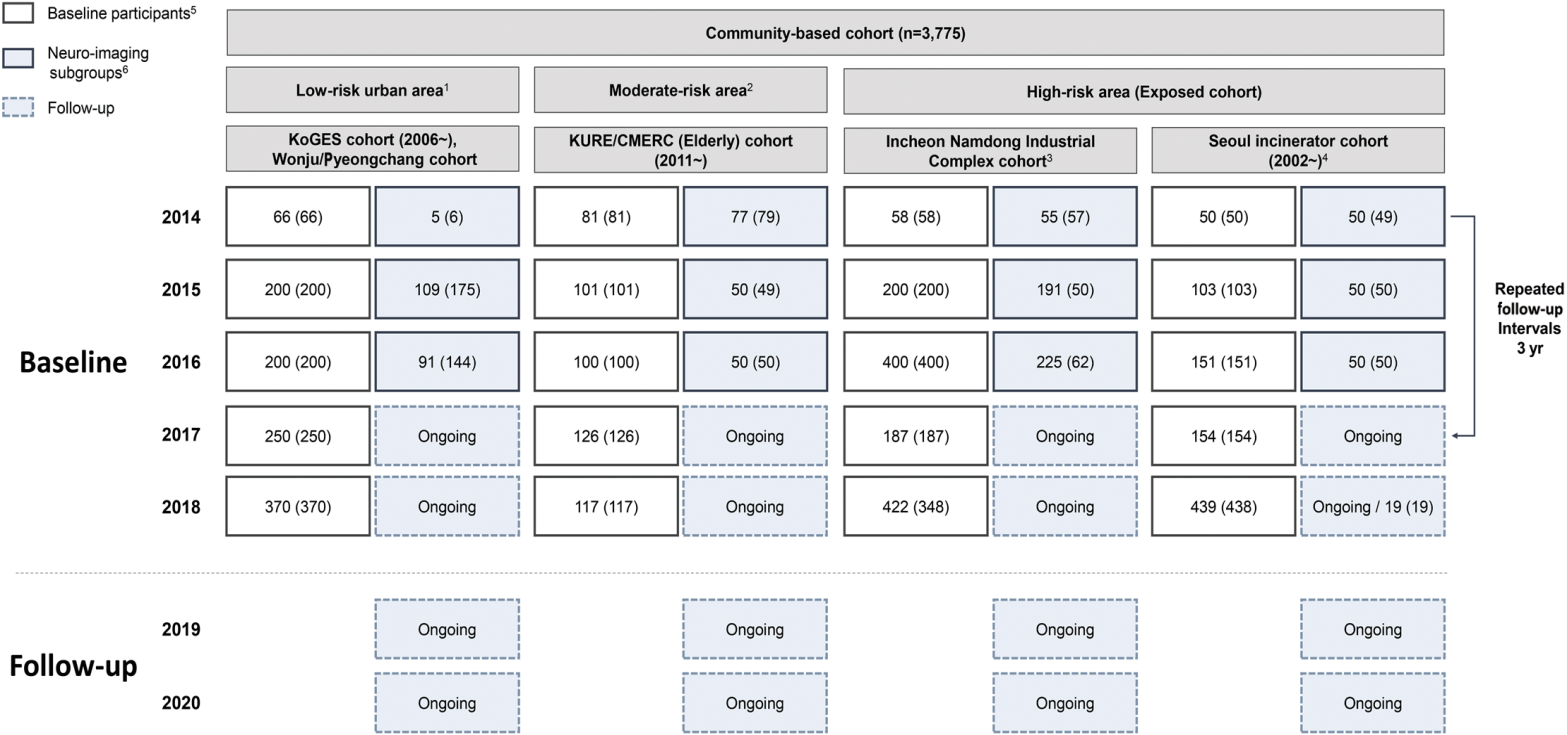
Flow diagram of baseline recruitment for the Environmental-Pollution-Induced Neurological EFfects (EPINEF) study. KoGES, Korean Genome and Epidemiology Study; KURE, Korean Urban Rural Elderly; CMERC, Cardiovascular and Metabolic Diseases Etiology Research Center; SNSB: Seoul Neuropsychological Screening Battery. ^1^Gangwha-gun, Wonju-si, Pyeongchang-si. ^2^Three administrative regions in Seoul. ^3^Incheon, a major city with the highest air pollutants levels. ^4^Three administrative regions (‘gu’) having major incinerator in Seoul. ^5^Survey number (biological specimen). ^6^Magnetic resonance imaging examined subjects of sub-groups (SNSB test examined subjects).

The EPINEF study was based on 6 prospective community-based cohorts (4 pre-existing and 2 newly established cohorts) involving areas with low, moderate, and high exposure risk (Figure 1). The 4 pre-existing cohorts included the Korean Genome and Epidemiology Study (KoGES) in Gangwha [10], the Korean Urban Rural Elderly cohort in Seoul [11], the Cardiovascular and Metabolic Diseases Etiology Research Center cohort in Seoul [12], and the Seoul incinerator cohort [13]. The new cohorts were based in Namdong-gu, Incheon (where a large industrial complex is located) and Wonju/Pyeongchang (rural areas). The Seoul incinerator and Namdong cohorts, which include regions within a specific distance (300 m or 1 km) from the source of pollution (industrial complex region) were classified as having high exposure risk, whereas the KoGES and Wonju/Pyeongchang cohorts, which are considered as rural areas, were classified to have low exposure risk. The remaining cohorts were classified to have moderate exposure risk. By distinguishing between high exposure risk and moderate/low exposure risk based on the surrounding proximity of the industrial complex, we were able to obtain a basis for the classification of exposure and non-exposure to environmental pollutants and analyze differences between exposed and non-exposed regions.

This multi-city design was chosen to obtain sufficient variation in the level of exposure to environmental pollutants. The survey centers were located at 3 university-based hospitals: Yonsei University Severance Hospital (Seoul and Gangwha), Gachon University Gil Medical Center (Incheon), and Wonju Severance Christian Hospital (Wonju and Pyeongchang).

### Eligibility and enrolment

**Figure 2.**
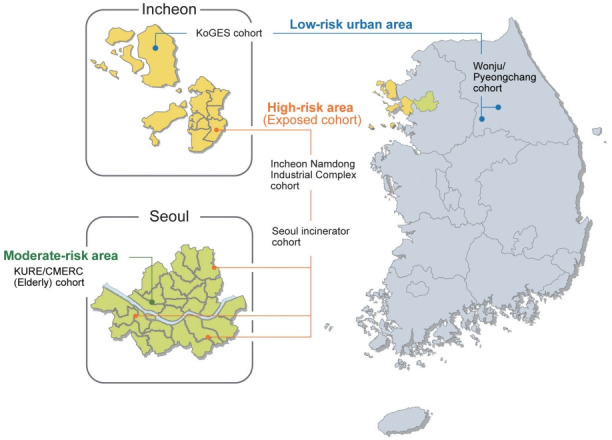
Geographical location of the survey sites of the Environmental-Pollution-Induced Neurological EFfects (EPINEF) study. KoGES, Korean Genome and Epidemiology Study; KURE, Korean Urban Rural Elderly; CMERC, Cardiovascular and Metabolic Diseases Etiology Research Center.

The eligibility criteria were age ≥ 50 years and no self-reported history of dementia, movement disorders, or stroke. Recruitment was conducted during the baseline survey period from August 2014 to March 2018. Eligible participants were asked to participate through on-site invitations, telephone calls, and media campaigns. Individuals who agreed to participate were invited to visit each survey center (Figure 2) and undergo questionnaire surveys, anthropometric measurements, blood sampling, and urine collection. Additionally, a total of 1,022 participants who visited between 2014 and 2018 were enrolled in a sub-cohort for brain magnetic resonance imaging (MRI) and detailed neuropsychological tests. We used competitive enrollment to achieve the goal of 50 participants per institution (approximately 250 participants per year). All participants were informed of the sub-cohort examination and voluntarily agreed to participate in the study. Although some factors (e.g., mean age, sex, education level, marriage status, smoking, alcohol consumption) significantly differed between the study participants included in the sub-cohort and those who were not, no significant differences were found in monthly income and cognitive function (Supplementary Material 1).

### Follow-up

The EPINEF study was designed as a longitudinal study, and the first follow-up surveys and environmental pollutant measurements will start in 2022, 8 years after the cohort was launched. A follow-up study was conducted in participants who had not changed their residence since the initial participation. Physical measurements, health surveys, laboratory tests, cognitive decline screening tests, and environmental pollutant measurements were performed using the same protocol as before. Medical history and events such as diseases and deaths that occurred between the baseline and follow-up surveys will be identified using the national health information database from the National Health Insurance Service. Follow-up of the neuroimaging sub-cohort is being conducted every 3 years to 4 years. To minimize loss to follow-up, we maintain regular personal contact through phone calls, e-mails, and post mails.

### Ethics statement

The EPINEF study was approved by the Yonsei University Health System Institutional Review Board (approval No. 4-2014-0359), and all participants provided written informed consent. The study was performed in accordance with the ethical standards as laid down in the 1964 Declaration of Helsinki and its later amendments or comparable ethical standards.

## MEASUREMENTS

### Data collection

The database consists of the following datasets: demographics and anthropometrics, laboratory test results (blood and urine), blood heavy metal concentrations, urine polycyclic aromatic hydrocarbon (PAH) metabolites, air pollutant concentrations, brain MRI data, and neuropsychological test results. These datasets can be linked at the individual level using encrypted identifiers. We also collected and stored DNA samples from all participants to investigate genetic-environmental interactions. Preliminarily, we performed a test to detect apolipoprotein E ε4 gene, which is associated with the risk of AD, using a subsample of 300 participants of the Namdong cohort. The baseline measurement items and the characteristics of all participants are shown in Tables 1 and 2, respectively.

### Environmental hazardous pollutants

From 2014 to 2017, we measured levels of indoor (window or veranda; 251 measurement points) and outdoor (points within the same residential complex or within 1 km; 208 measurement points) environmental pollutants in the participants’ residences (Supplementary Material 2). We investigated the presence of PM (10 μm or less in diameter [PM_10_], 2.5 μm or less in diameter [PM_2.5_]), total volatile organic compounds (including 17 different volatile organic compounds [VOCs]), PAHs (8 compounds), organophosphorus pesticides (6 types), and carbonyl compounds (10 compounds) in the environmental atmosphere. Further, we measured the concentration of heavy metals (19 metals) and pesticides in the total concentration of PM_10_ and PM_2.5_ (Supplementary Material 3). In addition, 301 consecutive indoor and outdoor measurements were conducted from 2016 to 2018 to adjust for temporal trends in prediction modeling of environmental pollutant exposure. Lastly, to identify the levels of VOCs, organophosphorus pesticides, and PAHs in the residences’ floor dust and translate these data to an inhalation exposure scenario, 84 field samplings were conducted in 2016 (Supplementary Material 2).

### Prediction of long-term concentrations of environmental hazardous pollutants

We developed prediction models for exposure to 10 pollutants: PM (PM_10_, PM_2.5_), manganese (in PM_10_ and PM_2.5_), acetaldehyde, styrene, toluene, and xylene (o-xylene, m, p-w xylene). Propionaldehyde, dichlorvos, tetrachloroethylene, and naphthalene showed multiple missing values or significantly low concentrations; thus, they were excluded from model development. As the sampling design of the EPINEF study is temporally imbalanced, we used additional air quality regulatory monitoring to account for temporal trends.

Due to the temporally-adjusted data, we applied the universal kriging model [14,15], a widely used prediction model for air pollution epidemiology, to estimate individual-level long-term air pollution concentrations at participants’ homes. This model assumes that pollutant concentration is composed of a long-term mean and spatial correlation [14,16,17]. The long-term mean was modeled based on a few summary predictors estimated from 320 geographic variables that represent potential pollution sources such as traffic and land use [18].

Finally, we derived 3 models for PM: (1) 2014-2017 multi-year average model, (2) 2016-2017 2-year average model, and (3) 2014- 2017 season average model. The final model had the best predictive power for measurement values. According to the cross-validation results, we selected the 2014-2017 multi-year average model for PM_10_, and the 2016-2017 2-year average model for PM_2.5_. For other pollutants, the final model was an annual average exposure prediction model based on a seasonal exposure model. We confirmed the power of the model using a cross-validation test. The concentrations of modeled environmental pollutants in each region are shown in Table 3.

### Polycyclic aromatic hydrocarbon metabolites in urine

To assess the exposure to environmental PAHs, we measured urinary concentrations of PAHs metabolites [19]. Urine samples were analyzed in a single laboratory (Green Cross Laboratories Corp., Yongin, Korea) using a gas chromatograph–mass spectrometer [20]. Urinary concentrations of the following four PAH metabolites were obtained: 1-hydroxypyrene (1-OHP), 2-naphthol, 1-hydroxyphenanthrene, and 2-hydroxyfluorene.

### Neuropsychological assessment

All baseline participants were screened for cognitive impairment [21] and depressive symptoms of dementia [22] using the Korean version of the Mini-Mental State Examination [23] and short form Korean version of the Geriatric Depression Scale (SGDS-K) [24]. Participants of the sub-cohort were examined using the Korean version of the Montreal Cognitive Assessment (K-MoCA) [25] and the Seoul Neuropsychological Screening Battery, second edition, which is a standardized neuropsychological test battery used in Korea consisting of 5 domains (Supplementary Material 4). Each measurement was a z-transformed score that was standardized based on the mean and standard deviation in the respective participant age-sex-education group [26].

### Acquisition and analysis of brain magnetic resonance imaging scans

The MRI scanners used in this study were Philips 3.0-Tesla Achieva MRI scanners (Yonsei University Severance Hospital and Wonju Severance Christian Hospital) or Siemens 3T Verio MRI scanners (Gachon University Gil Medical Center). The standardized brain MRI protocols included the following: diffusion tensor imaging (DTI), 3D-T1-magnetization-prepared-rapid-gradient-echo (3D-T1-MPRAGE), T2 turbo spin echo, resting-state functional MRI, and 3D T2 fluid-attenuated inversion recovery. Cortical thickness was evaluated using region of interest-based analyses and surfacebased analyses based on 3D T1 MPRAGE, and estimated using the FreeSurfer 6.0 pipeline (http://surfer.nmr.mgh.harvard.edu/). Owing to potential differences between the Philips and Siemens MRI scanners, 12 participants underwent brain MRI scans using both scanners. Brain cortical thickness, volume, and intracranial volume (ICV) measurements acquired with the Siemens scanner were transformed to equivalents of those acquired with the Philips scanner using linear regression models. The adjusted R-square values of the regression equations were 0.91 for cortical thickness, 0.93 for cortical volume, and 0.91 for ICV.

In addition, we have developed a biological brain age evaluation technology that can be used for early diagnosis and the prediction of dementia using brain MRI scans of the standard population and machine learning algorithms. We used this technique to estimate the brain age and dementia risk score of participants [27]. The MRI data and information collected in this study have been or will be uploaded to the Dementias Platform United Kingdom data portal established for dementia research [28].

## KEY FINDINGS AND PUBLICATIONS

### Air pollution, brain structural changes, and neuropsychological function

In a study published in 2020, we investigated the associations of ambient air pollutants (PM_10_, PM_2.5_, and nitrogen dioxide [NO_2_]) with brain MRI markers and neuropsychological function in adults [29]. An increase in PM_10_ or NO_2_ was associated with cortical thinning in the frontal and temporal lobes, and reduced volumes of the thalamus and nucleus accumbens. To minimize the impact of region-related confounding in air pollution exposure, we conducted propensity score matching (PSM) for the group exposed to high-level (66th percentile or higher) air pollution and the group exposed to low-level (33rd percentile or lower) air pollution. The propensity score was calculated using factors such as age, sex, educational level, marital status, income level, medical history, smoking, alcohol consumption, number of walking days (per week), body mass index (kg/m^2^), systolic blood pressure (mmHg), diastolic blood pressure (mmHg), total cholesterol level (mg/dL), and fasting blood glucose level (mg/dL). Even after PSM, the association between PM_10_ and temporal cortical thinning remained significant in both males and females. These findings suggest that subtle structural changes in the brain may be induced by exposure to airborne pollutants such as PM_10_, PM_2.5_, and NO_2_ in healthy adults. In addition, an increase in NO_2_ was associated with a lower K-MoCA score in both sexes. In females, an increase in all pollutants was associated with a lower z-score in the Ray Complex Figure Test delay function.

### Interaction of marriage in the relationship between air pollution and depressive symptoms

In another study, based on data from 2,122 married and 607 single participants, we studied the magnitude of the association between air pollutant exposure (PM_10_, PM_2.5_, and NO_2_) and SGDS-K scores according to marital status [30]. The analysis showed that married participants were less likely to have depressive symptoms associated with increased exposure to PM_10_ and PM_2.5_ concentrations than unmarried participants. In addition, the relationship between air pollution exposure and the SGDS-K score was analyzed after stratifying subjects by sex and region of residence. The stratification analysis found that married male and female who lived in non-metropolitan areas were less likely to have depressive symptoms associated with increased air pollutant concentrations than those who were not married. These findings suggest that marriage can act as a protective factor against depressive symptoms from exposure to air pollution.

### Urinary polycyclic aromatic hydrocarbons, brain structural changes, and neuropsychological function

A third study included 421 males and 528 females with brain MRI and PAH data [31]. We found that high concentrations of urinary 2-naphthol were associated with cortical thinning in the global, parietal, temporal, and insular lobes in males; this observation remained significant even after correction for multiple comparisons. In females, high 1-OHP concentrations were associated with frontal and parietal thinning and reduction of the pallidum volume. In males, it was associated with reduction of the caudate volume. In both sexes, scores of verbal learning and memory tests declined with increasing urinary 1-OHP concentrations.

## STRENGTHS AND WEAKNESSES

To the best of our knowledge, ours is the first cohort that has been specifically designed to comprehensively explore environmental pollutants related to neuroimaging and cognitive measures. This multicenter study has used a range of tools for quality control, including training for standardized protocols, regular research committee meetings, and frequent inspection of collected data. Moreover, data from nearly 1,000 brain 3T MRI examinations were collected at baseline, allowing us to conduct a relatively large neuroimaging study. This study is also strengthened by the availability of data on a variety of environmental pollutants using blood (heavy metals) and urine analyses (PAHs) and air pollution modeling (PM_10_, PM_2.5_, and NO_2_).

However, there are several limitations to be acknowledged. Firstly, given that neurodegeneration gradually progresses over a long time, it is impossible to estimate the risk of developing a neurodegenerative disease attributable to environmental pollution at this early stage. A long follow-up period would be needed to detect the association between environmental pollution and the incidence of dementia in our cohort. Nevertheless, we aimed to investigate brain MRI markers and neuropsychological performance as the primary endpoints, and this approach may be useful to improve the understanding of the evolution of neurodegeneration induced by environmental pollution. Secondly, information on exposure routes was not available for some of the pollutants. For example, we could not differentiate the effects of inhaled versus ingested PAHs because we measured PAHs in urine samples. Additional modeling for airborne PAHs may be necessary to address this issue. Lastly, selection bias may have occurred due to loss to follow-up in this cohort study involving repeated measurements of exposures or intermediate outcomes. To minimize this bias, we plan to maintain a high follow-up rate (≥ 80%). In addition, information about disease events in participants who failed to be followed up will be collected via telephone contacts and secondary data linkage.

## DATA ACCESSIBILITY

The datasets generated this study are not publicly available but are available from the corresponding author on reasonable request. The EPINEF research committee requests a short research proposal, including background information, research questions, methods, and authorship. The EPINEF research committee is responsible for the distribution and control of the data. Further information is available by contacting Professor Changsoo Kim, the principal investigator [preman@yuhs.ac].

## Figures and Tables

**Table 1.** Elements evaluated at baseline (2014-2018) in the Environmental-Pollution-Induced Neurological EFfects (EPINEF) cohort

Classification		Contents	Method
Physical measurements		Anthropometric measures (height, weight, body mass index, waist circumference^1^, hip circumference^1^, thigh circumference^1^), handgrip strength^2^, blood pressure	Examination
Health survey			Questionnaire/interview
	Demographic characteristics	Age, sex, date of birth (legal and actual), educational level, marriage status, household income, number of household members, period of residence, occupation, period of occupation, medical history of angina pectoris/ myocardial infarction, hypertension, dyslipidemia, diabetes mellitus, asthma, chronic obstructive pulmonary disease, arthritis, osteoporosis, cataract, glaucoma, mental health disease, depression, neoplasm, immediate family history of dementia, stroke, or Parkinson's disease, angina pectoris, diabetes mellitus, hypertension, mental health disease, neoplasm, reproductive health information (menarche, pregnancy, gestational diabetes and hypertension, oral contraceptive use, female hormone use), falls and external injuries (experiences/counts of falls and external injuries, injured areas, progress of hospital visits)	
	Quality of sleep	Pittsburgh Sleep Quality Index	
	Characteristics of environment surrounding the residence	Time spent indoors/outdoors, number of working days, ventilation availability, ventilation time, when cooking food (ventilation availability, fan operation), cleaning frequency, cleaning method, residence characteristics (regional scale, shape, area, number of building floors), highways around the residence (presence, traffic volume), beltway/main road near the residence (presence, traffic volume), characteristics of the residence, no. of roads around the residence, presence of facilities around the residence (bus stops, taxi stops, factories, incinerating facilities, gas stations/refueling stations, gasometer)	
	Quality of life	Subjective perception of health, life satisfaction, exercise ability, self-management, daily activities, pain, and anxiety	
	Health-related behaviors	Smoking, drinking, physical activity (vigorous-intensity exercise, moderate-intensity exercise, walking), group activities (religion, friendship, exercise/leisure, charity)	
Screening test			Questionnaire/self-report
	Cognitive function	Mini Mental State Examination-K, Korean-Montreal Cognitive Assessment	
	Mental health questionnaire	Depression (Korean version of the short form of Geriatric Depression Scale)	
Biochemical indicators		Laboratory analysis	
	Diagnostic blood test (blood)	WBC count, lymphocyte, monocyte, eosinophil, and basophil counts, RBC count, hemoglobin, hematocrit, MCV, MCH, MCHC, platelet count	
	Electrolytes (blood)	Ca, Cl, P	
	Lipid markers (blood)	Total cholesterol, triglyceride, HDL cholesterol	
	Hepatobiliary function test (blood)	Total protein, albumin, total bilirubin, bilirubin, ALP, AST, GGT, ALT	
	Hormone test (blood)	T3, TSH, Free T4, PTH-intact	
	Urinalysis (urine)	Nitrite, pH, urine protein, ketone	
	Renal function test (urine)	BUN, microalbumin, creatinine, uric acid	
	Other test (blood)	HbA1c, fasting blood glucose, hsCRP, vitamin D	
Environmental pollutants			
	Blood heavy metals concentration	Al, Hg, Pb, Mn	Laboratory analysis
	Urine PAH metabolites	2-OHF, 1-OHPHE, 1-OHP, 2-naphthol	Laboratory analysis
	Ambient air pollutants	PM_10_, PM_2.5_, Mn_10_ (Mn in PM_10_), Mn_2.5_ (Mn in PM_2.5_), acetaldehyde, styrene, toluene	Predictive modelling
Brain MRI^3^		Risk score of Alzheimer's disease, white matter hyperintensity, resting functional MRI, dDTI axial, faDTI axial, isoDTI axial, 3D T1WI non-contrast coronal, 3D T1WI non-contrast axial, 3D T1WI non-contrast sagittal, T2WI axial, 3D T2 fluid-attenuated inversion-recovery	Examination
Neuropsychological examination (SNSB)^3^		Memory function (SVLT recognition, SVLT free/delayed recalls, RCFT free/delayed recalls), language and related function (K-BNT), visuospatial function (RCFT copy), frontal/executive function (Stroop test-color reading, trail making test, COWAT, category fluency test, COWAT letter [phonemic] fluency test), attention (digit span test)	Examination

WBC, white blood cell count; RBC, red blood cell count; MCV, mean corpuscular volume; MCH, mean corpuscular hemoglobin; MCHC, mean corpuscular hemoglobin concentration; Ca, calcium; Cl, chloride; P, phosphate; HDL, high-density lipoprotein; ALP, alkaline phosphatase; AST, aspartate aminotransferase; GGT, gamma-glutamyl transferase; ALT, alanine transaminase; TSH, thyroid-stimulating hormone; PTH, parathyroid hormone; BUN, blood urea nitrogen; HbA1c, hemoglobin A1c; hs-CRP, high-sensitivity C-reactive protein; PAH, polycyclic aromatic hydrocarbon; Al, aluminum; Hg, Mercury; Pb, lead; Mn, manganese; 2-OHF, 2-hydroxyfluorene; 1-OHPHE, 1-hydroxyphenanthrene; 1-OHP, 1-hydroxypyrene; PM_10_, particulate matter 10 μm or less in diameter; PM_2.5_, particulate matter 2.5 μm or less in diameter; Mn_10_, manganese in PM_10_; Mn_2.5_, manganese in PM_2.5_; MRI, magnetic resonance imaging; SNSB, Seoul Neuropsychological Screening Battery; SVLT, Seoul Verbal Learning Test); RCFT, Ray Complex Figure Test; K-BNT, Korean-Boston Naming Test; COWAT, Controlled Oral Word Association Test.

1 Conducted from 2016.

2 Conducted from 2018.

3 Neuroimaging sub-cohort.

**Table 2.** Baseline characteristics of participants of the Environmental-Pollution-Induced Neurological EFfects (EPINEF) study according to the community-based cohorts

Characteristics			Total (n=3,775)	Unexposed cohort (n=1,611)		Exposed cohort (n=2,164)	
				Low-risk urban area	Moderate-risk area	High-risk area	
				KoGES cohort, Wonju/Pyeongchang cohort	KURE/CMERC cohort	Incheon Namdong Industrial Complex cohort	Seoul incinerator cohort
Age (yr)			68.80±6.67	66.49±7.48	73.95±4.83	68.94±5.74	68.39±6.12
Sex							
	Male		1,209 (32.0)	463 (38.3)	187 (15.5)	343 (28.4)	216 (17.9)
	Female		2,566 (68.0)	623 (24.3)	338 (13.2)	924 (36.0)	681 (26.5)
Education period (yr)			12.00 (9.00-14.00)	12.00 (6.00-14.00)	12.00 (9.00-14.00)	12.00 (9.00-14.00)	12.00 (9.00-14.00)
Questionnaires							
	Marital status						
		Marriage, married and living with a spouse	2,897 (76.7)	913 (31.5)	382 (13.2)	934 (32.2)	668 (23.1)
		Not married	801 (21.2)	106 (13.2)	142 (17.7)	329 (41.1)	224 (28.0)
		Unknown	77 (2.0)	67 (87.0)	1 (1.3)	4 (5.2)	5 (6.5)
	Monthly income (104 Korean won)						
		<100	856 (22.7)	285 (33.3)	148 (17.3)	252 (29.4)	171 (20.0)
		100-199	932 (24.7)	286 (30.7)	134 (14.4)	346 (37.1)	166 (17.8)
		200-299	651 (17.2)	155 (23.8)	74 (11.4)	249 (38.2)	173 (26.6)
		300-399	443 (11.7)	140 (31.6)	38 (8.6)	150 (33.9)	115 (26.0)
		≥400	508 (13.5)	173 (34.1)	30 (5.9)	154 (30.3)	151 (29.7)
		Unknown	385 (10.2)	47 (12.2)	101 (26.2)	116 (30.1)	121 (31.4)
	Smoking status						
		Non-smokers	2,872 (76.1)	778 (27.1)	383 (13.3)	986 (34.3)	725 (25.2)
		Ex-smokers	727 (19.3)	251 (34.5)	121 (16.6)	219 (30.1)	136 (18.7)
		Current smokers	176 (4.7)	57 (32.4)	21 (11.9)	62 (35.2)	36 (20.4)
	Drinking status						
		Non-drinkers	2,051 (54.3)	610 (29.7)	289 (14.1)	713 (34.8)	439 (21.4)
		Ex-drinkers	259 (6.9)	58 (22.4)	59 (22.8)	110 (42.5)	32 (12.4)
		Current drinkers	1,465 (38.8)	418 (28.5)	177 (12.1)	444 (30.3)	426 (29.1)
Anthropometric measurements							
	Height (cm)		157.44±10.67	158.40±13.17	157.17±7.82	156.97±11.09	157.09±7.67
	Weight (kg)		61.27±9.89	62.27±11.49	60.30±9.00	61.32±9.65	60.56±8.43
	Body mass index (kg/m^2^)		24.58±3.41	24.53±3.87	24.39±3.03	24.73±3.49	24.53±2.89
	Systolic blood pressure (mmHg)		130.00 (120.00-140.00)	125.00 (116.00-134.00)	128.50 (117.50-140.50)	132.00 (124.00-141.00)	132.00 (121.00-141.00)
	Diastolic blood pressure (mmHg)		76.00 (70.00-81.00)	79.00 (70.00-82.00)	73.00 (67.50-79.00)	76.00 (70.00-81.00)	75.00 (70.00-81.00)
Clinical examination							
	Fasting blood glucose (mg/dL)		92.00 (85.00-99.00)	94.00 (88.00-102.00)	93.00 (87.00-102.00)	89.00 (83.00-98.00)	90.00 (84.00-97.00)
	HbA1c (%)		5.70 (5.50-6.00)	5.60 (5.40-5.90)	5.80 (5.50-6.10)	5.70 (5.50-6.00)	5.70 (5.50-5.90)
	Total cholesterol (mg/dL)		183.00 (158.00-207.00)	187.00 (162.00-210.00)	176.00 (153.00-200.00)	181.00 (157.00-204.00)	184.00 (160.00-208.00)
	HDL cholesterol (mg/dL)		51.00 (44.00-59.00)	50.00 (42.00-59.00)	50.00 (43.00-58.00)	51.00 (44.00-59.00)	53.00 (46.00-61.00)
	Triglycerides (mg/dL)		112.00 (83.00-146.00)	116.00 (86.00-153.00)	113.00 (84.00-142.00)	111.00 (83.00-145.00)	105.00 (80.00-144.00)
	BUN (mg/dL)		15.90 (13.60-18.60)	15.90 (13.60-18.50)	16.65 (14.20-19.30)	15.70 (13.40-18.50)	15.80 (13.50-18.50)
	Creatinine (mg/dL)		0.80 (0.67-0.93)	0.82 (0.68-0.96)	0.87 (0.74-0.99)	0.80 (0.68-0.92)	0.74 (0.64-0.88)
	AST (IU/L)		25.00 (22.00-29.00)	26.00 (23.00-30.00)	25.00 (22.00-28.00)	26.00 (23.00-30.00)	25.00 (22.00-29.00)
	ALT (IU/L)		20.00 (17.00-25.00)	20.00 (17.00-26.00)	19.00 (15.00-24.00)	21.00 (17.00-25.00)	20.00 (17.00-25.00)
	hs-CRP (mg/L)		0.67 (0.41-1.19)	0.70 (0.43-1.21)	0.64 (0.41-1.16)	0.68 (0.41-1.20)	0.64 (0.40-1.14)
Cognitive function^1^							
	Normal		3,422 (90.6)	994 (29.0)	388 (11.3)	1,199 (35.0)	841 (24.6)
	Mild cognitive impairment		273 (7.2)	75 (27.5)	106 (38.8)	50 (18.3)	42 (15.4)
	Severe cognitive impairment		80 (2.1)	17 (21.2)	31 (38.7)	18 (22.5)	14 (17.5)
Depression^2^							
	Normal		3,210 (88.2)	955 (29.7)	379 (11.8)	1.086 (33.8)	790 (24.6)
	Depression		428 (11.8)	93 (21.7)	130 (30.4)	133 (31.1)	72 (16.8)

Values are presented as mean±standard deviation, number (%), or median (minimun-maximun). KoGES, Korean Genome and Epidemiology Study; KURE, Korean Urban Rural Elderly; CMERC, Cardiovascular and Metabolic Diseases Etiology Research Center; HbA1c, glycated hemoglobin; HDL, high-density lipoprotein; BUN, blood urea nitrogen; AST, aspartate transaminase; ALT, alanine aminotransferase; hs-CRP, high-sensitivity C-reactive protein.

1 Severe cognitive impairment was defined as a Mini-Mental State Examination (Korean version, MMSE-K) screening test score ≤19; mild cognitive impairment was defined as a MMSE-K screening test score of 20-23.

2 Depression was defined as a Geriatric Depression Scale (short form, Korean version) screening test score <7.

**Table 3.** Concentration of environmental pollutants in participants of the Environmental-Pollution-Induced Neurological EFfects (EPINEF) study according to the community-based cohorts

Variables	Total (n=3,775)	Unexposed cohort (n=1,611)		Exposed cohort (n=2,164)	
		Low-risk urban area	Moderate-risk area	High-risk area	
		KoGES cohort, Wonju/Pyeongchang cohort	KURE/CMERC cohort	Incheon Namdong Industrial Complex cohort	Seoul incinerator cohort
PM_10_ (µg/m^3^)	45.90±2.30	42.72±1.52	46.81±1.21	47.32±0.77	46.98±1.26
PM_2.5_ (µg/m^3^)	26.13±0.58	26.66±0.26	25.84±0.54	25.89±0.49	26.03±0.60
NO_2_ (ppb)^1^	27.68±10.71	10.91±3.56	33.72±5.08	33.34±3.34	33.75±4.26
Mn_10_ (ng/m^3^)	12.20±2.67	9.34±2.18	12.45±1.88	13.67±1.79	13.23±2.03
Mn_2.5_ (ng/m^3^)	10.59±1.76	12.94±0.79	9.21±1.09	9.90±0.99	9.70±1.05
Acetaldehyde (µg/m^3^)	10.72±1.58	8.73±1.36	11.09±0.75	11.88±0.73	11.12±0.65
Styrene (µg/m^3^)	16.30±4.21	13.01±2.57	16.80±3.65	17.55±4.22	17.99±3.94
Toluene (µg/m^3^)	32.97±5.54	26.57±3.59	32.76±3.15	36.97±3.99	34.71±3.42

Values are presented as mean±standard deviation. KoGES, Korean Genome and Epidemiology Study; KURE, Korean Urban Rural Elderly; CMERC, Cardiovascular and Metabolic Diseases Etiology Research Center; PM_10_, particulate matter 10 μm or less in diameter; PM_2.5_, particulate matter 2.5 μm or less in diameter; NO_2_, nitrogen dioxide; Mn_10_, manganese in PM_10_; Mn_2.5_, manganese in PM_2.5_.

1 The measurements were taken in the first to third year: Total number (n=1,711); Unexposed cohort (n=749); Exposed cohort (n=962).

## References

[1] Prince M, Wimo A, Guerchet M, Ali G, Wu Y, Prina M World Alzheimer report 2015: the global impact of dementia. https://www.alzint.org/u/WorldAlzheimerReport2015.pdf.

[2] Prince M, Bryce R, Albanese E, Wimo A, Ribeiro W, Ferri CP (2013). The global prevalence of dementia: a systematic review and meta-analysis. Alzheimers Dement.

[3] Wu YT, Beiser AS, Breteler MM, Fratiglioni L, Helmer C, Hendrie HC (2017). The changing prevalence and incidence of dementia over time - current evidence. Nat Rev Neurol.

[4] GBD 2016 DALYs and HALE Collaborators (2017). Global, regional, and national disability-adjusted life-years (DALYs) for 333 diseases and injuries and healthy life expectancy (HALE) for 195 countries and territories, 1990-2016: a systematic analysis for the Global Burden of Disease Study 2016. Lancet.

[5] Kelley AS, McGarry K, Gorges R, Skinner JS (2015). The burden of health care costs for patients with dementia in the last 5 years of life. Ann Intern Med.

[6] Wong W (2020). Economic burden of Alzheimer disease and managed care considerations. Am J Manag Care.

[7] Baek JY, Lee E, Jung HW, Jang IY (2021). Geriatrics fact sheet in Korea 2021. Ann Geriatr Med Res.

[8] Kim YJ, Han JW, So YS, Seo JY, Kim KY, Kim KW (2014). Prevalence and trends of dementia in Korea: a systematic review and meta-analysis. J Korean Med Sci.

[9] Park JH, Eum JH, Bold B, Cheong HK (2013). Burden of disease due to dementia in the elderly population of Korea: present and future. BMC Public Health.

[10] Kim Y, Han BG, KoGES group (2017). Cohort profile: the Korean Genome and Epidemiology Study (KoGES) consortium. Int J Epidemiol.

[11] Lee EY, Kim HC, Rhee Y, Youm Y, Kim KM, Lee JM (2014). The Korean urban rural elderly cohort study: study design and protocol. BMC Geriatr.

[12] Shim JS, Song BM, Lee JH, Lee SW, Park JH, Choi DP (2019). Cohort profile: the Cardiovascular and Metabolic Diseases Etiology Research Center cohort in Korea. Yonsei Med J.

[13] Leem JH, Hong YC, Lee KH, Kwon HJ, Chang YS, Jang JY (2003). Health survey on workers and residents near the municipal waste and industrial waste incinerators in Korea. Ind Health.

[14] Sampson PD, Richards M, Szpiro AA, Bergen S, Sheppard L, Larson TV (2013). A regionalized national universal kriging model using Partial Least Squares regression for estimating annual PM_2.5_ concentrations in epidemiology. Atmos Environ (1994).

[15] Kim SY, Sheppard L, Bergen S, Szpiro AA, Sampson PD, Kaufman J (2013). Prediction of fine particulate matter chemical components for the Multi-Ethnic Study of Atherosclerosis cohort: a comparison of two modeling approaches. http://biostats.bepress.com/uwbiostat/paper398.

[16] Kim SY, Sheppard L, Bergen S, Szpiro AA, Sampson PD, Kaufman JD (2016). Prediction of fine particulate matter chemical components with a spatio-temporal model for the Multi-Ethnic Study of Atherosclerosis cohort. J Expo Sci Environ Epidemiol.

[17] Kim SY, Olives C, Sheppard L, Sampson PD, Larson TV, Keller JP (2017). Historical prediction modeling approach for estimating long-term concentrations of PM_2.5_ in cohort studies before the 1999 implementation of widespread monitoring. Environ Health Perspect.

[18] Kim SY, Song I (2017). National-scale exposure prediction for long-term concentrations of particulate matter and nitrogen dioxide in South Korea. Environ Pollut.

[19] Scherer G, Frank S, Riedel K, Meger-Kossien I, Renner T (2000). Biomonitoring of exposure to polycyclic aromatic hydrocarbons of nonoccupationally exposed persons. Cancer Epidemiol Biomarkers Prev.

[20] Li Z, Sandau CD, Romanoff LC, Caudill SP, Sjodin A, Needham LL (2008). Concentration and profile of 22 urinary polycyclic aromatic hydrocarbon metabolites in the US population. Environ Res.

[21] Lin JS, O’Connor E, Rossom RC, Perdue LA, Burda BU, Thompson M (2013). Screening for cognitive impairment in older adults: an evidence update for the U.S. Preventive Services Task Force.

[22] Burke WJ, Houston MJ, Boust SJ, Roccaforte WH (1989). Use of the Geriatric Depression Scale in dementia of the Alzheimer type. J Am Geriatr Soc.

[23] Han C, Jo SA, Jo I, Kim E, Park MH, Kang Y (2008). An adaptation of the Korean mini-mental state examination (K-MMSE) in elderly Koreans: demographic influence and population-based norms (the AGE study). Arch Gerontol Geriatr.

[24] Bae JN, Cho MJ (2004). Development of the Korean version of the Geriatric Depression Scale and its short form among elderly psychiatric patients. J Psychosom Res.

[25] Kang Y, Park JS, Yu KH, Lee BC (2009). A reliability, validity, and normative study of the Korean-Montreal Cognitive Assessment (K-MoCA) as an instrument for screening of vascular cognitive impairment (VCI). Korean J Clin Psychol.

[26] Jahng S, Na DL, Kang Y (2015). Constructing a composite score for the Seoul neuropsychological screening battery-core. Dement Neurocogn Disord.

[27] Lee JS, Kim C, Shin JH, Cho H, Shin DS, Kim N (2018). Machine learning-based individual assessment of cortical atrophy pattern in Alzheimer’s disease spectrum: development of the classifier and longitudinal evaluation. Sci Rep.

[28] Bauermeister S, Orton C, Thompson S, Barker RA, Bauermeister JR, Ben-Shlomo Y (2020). The dementias platform UK (DPUK) data portal. Eur J Epidemiol.

[29] Cho J, Noh Y, Kim SY, Sohn J, Noh J, Kim W (2020). Long-term ambient air pollution exposures and brain imaging markers in Korean adults: the Environmental Pollution-Induced Neurological EFfects (EPINEF) study. Environ Health Perspect.

[30] Kim H, Cho J, Isehunwa O, Noh J, Noh Y, Oh SS (2020). Marriage as a social tie in the relation of depressive symptoms attributable to air pollution exposure among the elderly. J Affect Disord.

[31] Cho J, Sohn J, Noh J, Jang H, Kim W, Cho SK (2020). Association between exposure to polycyclic aromatic hydrocarbons and brain cortical thinning: the Environmental Pollution-Induced Neurological EFfects (EPINEF) study. Sci Total Environ.

