# Supplementary material for: Cohort profile: the Environmental-Pollution-Induced Neurological EFfects (EPINEF) study: a multicenter cohort study of Korean adults
Source: Epidemiol Health. 2021 Sep 16;43:e2021067. doi: 10.4178/epih.e2021067 (PMC8689119; doi:10.4178/epih.e2021067)
Supplement: Supplementary file 1 [file epih-43-e2021067-suppl1.docx]

**Supplementary Material 1.** Comparison of characteristics between study participants with and without brain MRI data.

| **Characteristics** | | **Excluded participants without brain MRI data** | **Neuroimaging sub-cohort** | **P-value** |
| --- | --- | --- | --- | --- |
|  |  | **(n=2,753)** | **(n=1,022)** |  |
| Age, mean (SD) | | 69.29 (6.71) | 67.49 (6.38) | <.0001 |
| Sex, n (%) | |  |  | <.0001 |
|  | Male | 759 (27.57) | 450 (44.03) |  |
|  | Female | 1,994 (72.43) | 572 (55.97) |  |
| Education period, mean (SD) | | 11.20 (4.69) | 10.14 (4.58) | <.0001 |
| Marriage status, n (%) | |  |  | <.0001 |
|  | Marriage, married and living with a spouse | 2,044 (74.25) | 853 (83.46) |  |
|  | Not married | 640 (23.25) | 161 (15.75) |  |
|  | Unknown (missing) | 69 (2.51) | 8 (0.78) |  |
| Monthly income (10,000 won), n (%) | |  |  | 0.07 |
|  | <100 | 646 (23.47) | 210 (20.55) |  |
|  | 100-199 | 645 (23.43) | 287 (28.08) |  |
|  | 200-299 | 483 (17.54) | 168 (16.44) |  |
|  | 300-399 | 328 (11.91) | 115 (11.25) |  |
|  | ≥ 400 | 370 (13.44) | 138 (13.50) |  |
|  | Unknown | 281 (10.21) | 104 (10.18) |  |
| Smoking status, n (%) | |  |  | <.0001 |
|  | Non-smokers | 2,184 (79.33) | 688 (67.32) |  |
|  | Ex-smokers | 452 (16.42) | 275 (26.91) |  |
|  | Current smokers | 117 (4.25) | 59 (5.77) |  |
| Drinking status, n (%) | |  |  | <.0001 |
|  | Non-drinkers | 1,577 (57.28) | 474 (46.38) |  |
|  | Ex-drinkers | 126 (4.58) | 133 (13.01) |  |
|  | Current drinkers | 1050 (38.14) | 415 (40.61) |  |
| Cognitive function, n (%)^1^ | |  |  | 0.39 |
|  | Normal | 2,489 (90.41) | 933 (91.29) |  |
|  | Mild cognitive impairment | 208 (7.56) | 65 (6.36) |  |
|  | Severe cognitive impairment | 56 (2.03) | 24 (2.35) |  |

*Footnotes.* Abbreviations: SD, standard deviation; MRI, Magnetic Resonance Imaging.

^1^Severe cognitive impairment was defined as a Mini-Mental State Examination (Korean version, MMSE-K) screening test score ≤ 19; mild cognitive impairment was defined as a MMSE-K screening test score of 20-23.
