# Supplementary material for: Cohort profile: the Environmental-Pollution-Induced Neurological EFfects (EPINEF) study: a multicenter cohort study of Korean adults
Source: Epidemiol Health. 2021 Sep 16;43:e2021067. doi: 10.4178/epih.e2021067 (PMC8689119; doi:10.4178/epih.e2021067)
Supplement: Supplementary file 2 [file epih-43-e2021067-suppl2.docx]

Supplementary Material 2. Measurements of indoor and outdoor environmental pollutants (2014-2018) according to the community-based cohorts included in the Environmental-Pollution-Induced Neurological Effects (EPINEF) consortium

| **Cohort** | **No of measurements** | **Airborne particle** | | | | **Floor dust** |
| --- | --- | --- | --- | --- | --- | --- |
|  |  | **Outdoor** | | **Indoor** | |  |
|  |  | **One time** | **Consecutive** | **One time** | **Consecutive** |  |
| Wonju-Pyeongchang | 229 | 39 | 67 | 39 | 67 | 17 |
| KoGES  (Incheon and Kwanghwa) | 27 | 20 | - | 7 | - | - |
| KURE | 114 | 47 | - | 47 | - | 20 |
| Seoul incinerator studies | 281 | 66 | 69 | 50 | 69 | 27 |
| Incheon Namdong industrial complex | 276 | 79 | 56 | 65 | 56 | 20 |
| Total | 927 | 251 | 192 | 208 | 192 | 84 |

*Footnotes.* Abbreviations: KoGES, Korean Genome and Epidemiology Study; KURE, Korean Urban Rural Elderly.
