# Supplementary material for: Cohort profile: the Environmental-Pollution-Induced Neurological EFfects (EPINEF) study: a multicenter cohort study of Korean adults
Source: Epidemiol Health. 2021 Sep 16;43:e2021067. doi: 10.4178/epih.e2021067 (PMC8689119; doi:10.4178/epih.e2021067)
Supplement: Supplementary file 3 [file epih-43-e2021067-suppl3.docx]

Supplementary Material 3. Environmental pollutants measured in the residences of participants of the Environmental-Pollution-Induced Neurological Effects (EPINEF) study (from 2014 to 2017)

| **Category** | **Environmental hazardous material** |
| --- | --- |
| Particulate matter | PM_10_, PM_2.5_ |
| Metal | Al, Ti, V, Cr, Mn, Co, Ni, Cu, Zn, Ge, As, Se, Sr, Mo, Ag, Cd, Sn, Sb, Pb |
| TVOCs | dichloromethane, methyl ethyl ketone, isobutyl ketone, chloroform, 1,2-dichloroethane, methyl isobutyl ketone, benzene, carbon tetrachloride, trichloroethylene, tetrachloroethylene, n-butyl acetate, toluene, butylbenzene, m,p-xylene, o-xylene, styrene, aniline |
| Carbonyl compounds | formaldehyde, acetaldehyde, acetone, acrolein, propionaldehyde, crotonaldehyde, butyraldehyde, benzaldehyde, isovaleraldehyde, valeraldehyde |
| PAHs | naphthalene, acenaphthylene, fluorene, phenanthrene, anthracene, fluoranthene, pyrene, benzo(a)anthracene, benzo(k)fluoranthene, benzo(a)pyrene, dibenz(a,h)anthracene, benzo(g,h,i)perylene, indeno(1,2,3,-cd)pyrene |
| Pesticides | dichlorvos, chlorpyrifos, diazinon, malathion, parathion, fenitrothion |

*Footnotes.* Abbreviations: PM, particulate matter; Al, aluminium; Ti, titanium; V, vanadium; Cr, chromium; Mn, manganese; Co, cobalt; Ni, nickel; Cu, copper; Zn, zinc; Ge, germanium; As, arsenic; Se, selenium; Sr, strontium; Mo, molybdenum; Ag, silver; Cd, cadmium; Sn, tin; Sb, antimony; Pb, lead; TVOCs, total volatile organic compounds; PAHs, polycyclic aromatic hydrocarbons.
