# Supplementary material for: Cohort profile: the Environmental-Pollution-Induced Neurological EFfects (EPINEF) study: a multicenter cohort study of Korean adults
Source: Epidemiol Health. 2021 Sep 16;43:e2021067. doi: 10.4178/epih.e2021067 (PMC8689119; doi:10.4178/epih.e2021067)
Supplement: Supplementary file 4 [file epih-43-e2021067-suppl4.docx]

Supplementary Material 4. List of tests and subtests included in the Seoul Neuropsychological Screening Battery used to assess the neuro-imaging sub-cohort of the Environmental-Pollution-Induced Neurological Effects (EPINEF) study

| **Category** | **Test** | **Subtest** |
| --- | --- | --- |
| Memory function | SVLT | SVLT recognition  SVLT immediate recall  SVLT free/delayed recalls |
|  | RCFT | RCFT immediate recall  RCFT free/delayed recall |
| Language and related functions | K-BNT | Short form of K-BNT |
| Visuospatial function | RCFT | RCFT copy |
| Frontal/Executive function | Stroop test-colour reading | Stroop test |
|  | Trail making test | Trail making test |
|  | COWAT | Semantic fluency test  Animal  Supermarket |
|  |  | Letter (phonemic) fluency test |
| Attention | Digit span test | Digit span test |

*Footnotes.* Abbreviations: SVLT, Seoul Verbal Leaning Test; RCFT, Ray Complex Figure Test; K-BNT, Korean-Boston Naming Test; RCFT, Ray Complex Figure Test; COWAT, Controlled Oral Word Association Test.
